# Supplementary material for: Insights into the Molecular Basis of Huanglongbing Tolerance in Persian Lime (Citrus latifolia Tan.) through a Transcriptomic Approach
Source: Int J Mol Sci. 2023 Apr 19;24(8):7497. doi: 10.3390/ijms24087497 (PMC10144405; doi:10.3390/ijms24087497)
Supplement: Supplementary file 1 [file ijms-24-07497-s001.zip › ijms-2270145-supplementary figures S1-S3.pdf]

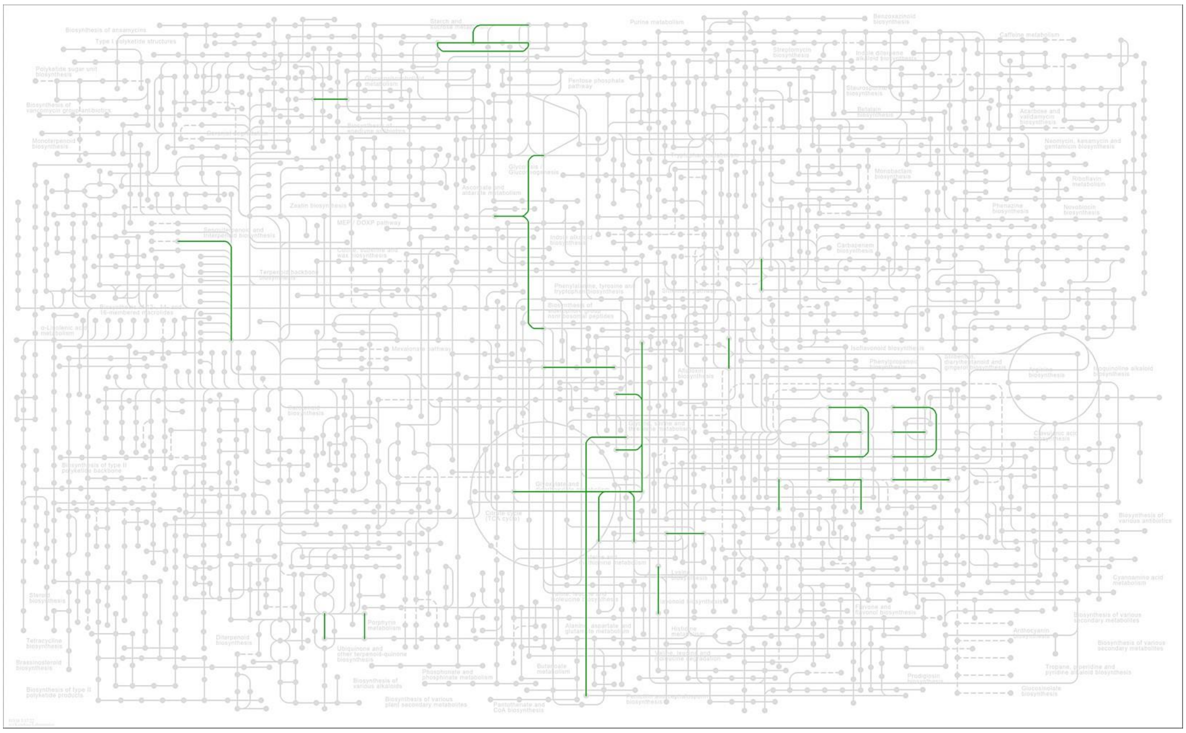

Figure S1. Metabolic pathways in HLB-asymptomatic leaves of Persian lime**.** Green lines indicate the differentially regulated pathways.

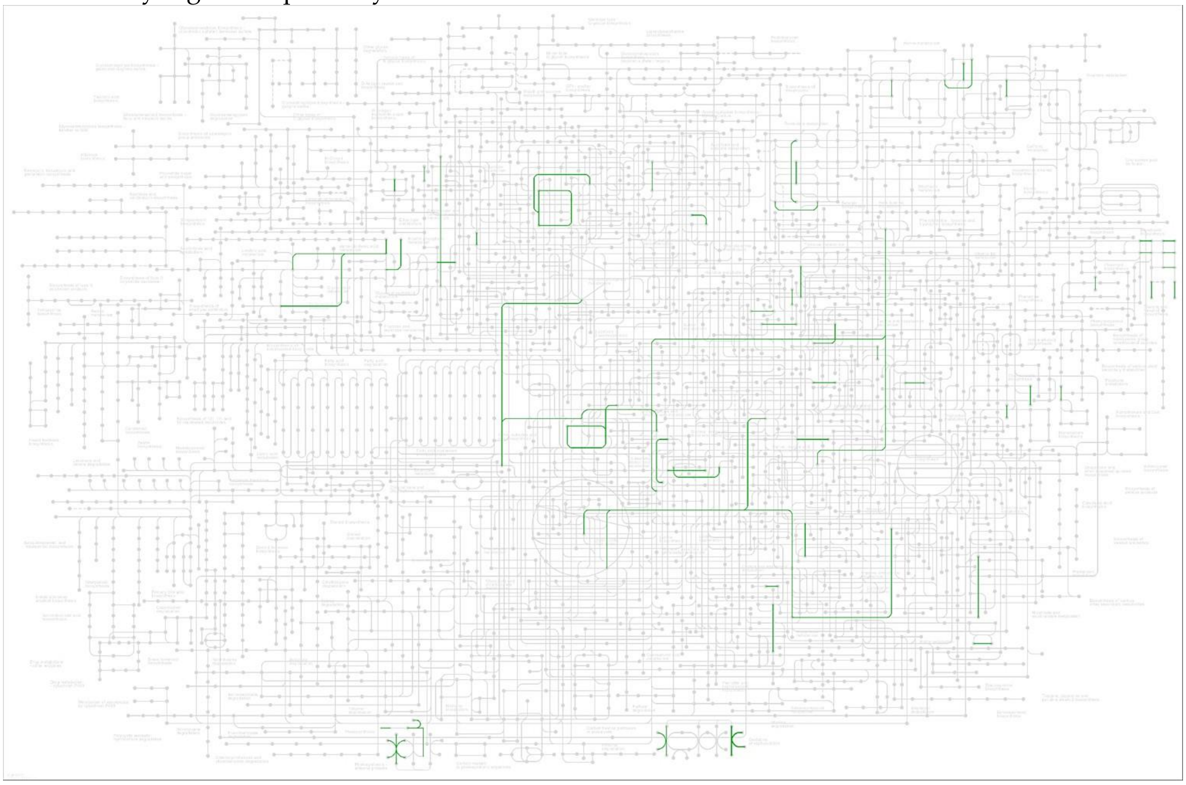

Figure S2. Metabolic pathways in HLB-symptomatic leaves of Persian lime**.** Green lines indicate the differentially regulated pathways.

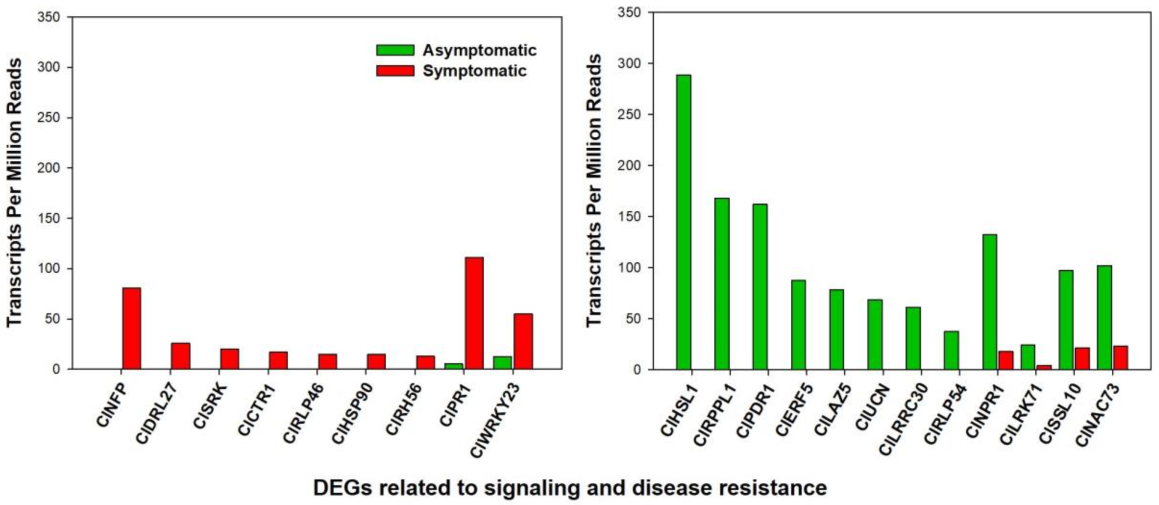

(**a**) (**b**)

Figure S3. Comparison of transcripts per million reads of DEGs related to signaling and disease resistance that was **a)** upregulated and **b)** downregulated in HLB-symptomatic leaves of Persian lime
